# Self‐Powered All‐in‐One Wearable Bioelectronics Driven by Pneumatic Energy Buffering of Daily Footsteps

**DOI:** 10.1002/advs.77158

**Published:** 2026-08-11

**Authors:** Ji‐Seok Kim, Tae Woog Kang, Hyunjoon Yoo, Jawon Ha, Yunuo Huang, Hee Han, Chi Won Ahn, Woon‐Hong Yeo, Il‐Kwon Oh

**Affiliations:** ^1^ Department of Mechanical Engineering Korea Advanced Institute of Science and Technology (KAIST) Daejeon Republic of Korea; ^2^ Department of Mechanical Engineering Hanbat National University Daejeon Republic of Korea; ^3^ George W. Woodruff School of Mechanical Engineering Georgia Institute of Technology Atlanta Georgia USA; ^4^ Wearable Intelligent Systems and Healthcare Center (WISH Center) At the Institute for Matter and Systems Georgia Institute of Technology Atlanta Georgia USA; ^5^ National Nano Fab Center (NNFC) Daejeon Republic of Korea; ^6^ Wallace H. Coulter Department of Biomedical Engineering Georgia Institute of Technology and Emory University School of Medicine Atlanta Georgia USA; ^7^ Parker H. Petit Institute For Bioengineering and Biosciences Georgia Institute of Technology Atlanta Georgia USA

**Keywords:** integrated biosensing, PPG sensors, self‐powered devices, smart shoes, wearable energy harvesters

## Abstract

Recent advances in wearable biosensing technologies have underscored the importance of continuous and reliable personal health monitoring. However, the limited size and capacity of conventional batteries remain a critical bottleneck for long‐term operation. Here, we present integrated self‐powered wearable bioelectronics driven by an air‐pumping pneumatic energy buffering mechanism, which converts intermittent human motion into sustained mechanical rotation during walking and efficiently generates electrical energy. The air‐driven rotation persists for 1.5 s per step, enabling continuous power generation from inherently low‐frequency biomechanical inputs and serving as a lightweight frequency‐regulating mechanism for wearable energy harvesters. To facilitate efficient integration with energy storage systems, a power management system is developed to directly charge a compact battery. In addition, a wearable photoplethysmography (PPG) device is designed to mitigate motion‐induced artifacts, enabling robust physiological signal acquisition during dynamic conditions. Owing to its Velcro‐type design, the PPG system demonstrates improved signal reliability compared to conventional devices. By integrating the energy harvester with the PPG, a self‐powered wearable biosensing platform has been successfully demonstrated. Unlike conventional wearable energy harvesters that directly convert transient biomechanical inputs into short‐duration electrical outputs, the proposed system introduces a pneumatic energy buffering mechanism that enables quasi‐continuous power generation from inherently intermittent human motion.

## Graphical abstract

A pneumatic energy‐buffering strategy transforms transient and intermittent footsteps into quasi‐continuous electrical energy for a self‐powered wearable biosensing platform. The footwear‐integrated air‐pumping harvester, combined with the rationally designed power‐management system, powers a wearable ankle PPG device with reduced motion artifacts, enabling real‐time all‐day SpO_2_ monitoring and offering a practical route toward autonomous wearable bioelectronics.

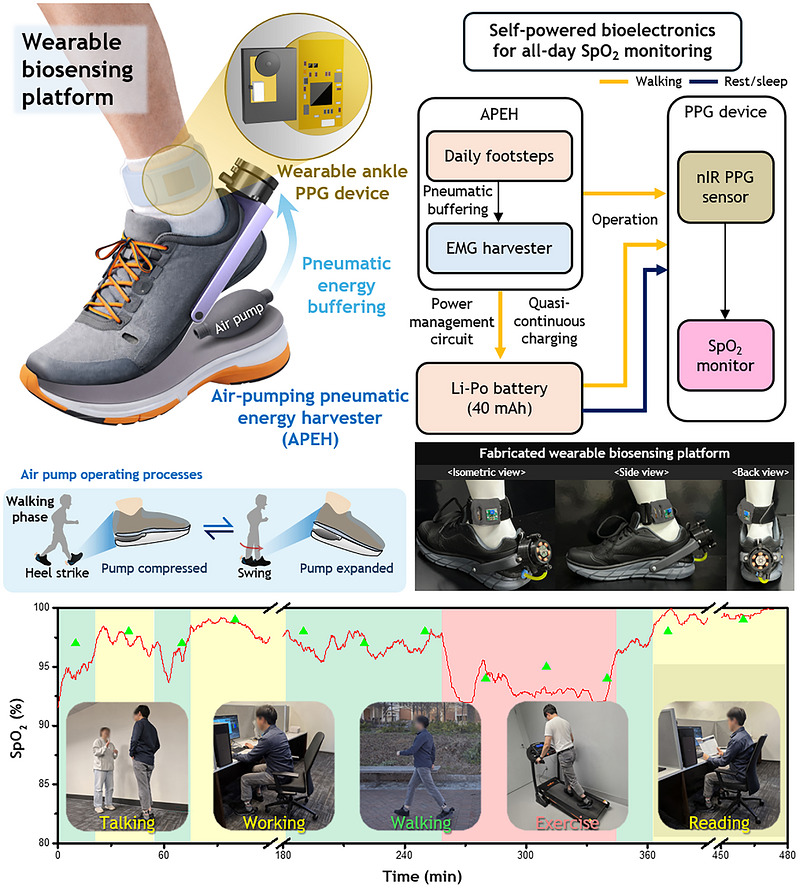

## 1 Introduction

Recent advances in wearable electronics and biosensor technologies have enabled continuous monitoring of physiological signals, significantly expanding the role of personal healthcare systems from intermittent measurement to long‐term health tracking [1, 2]. Wearable devices equipped with photoplethysmography (PPG), electrocardiography (ECG), and motion sensors are increasingly used for real‐time assessment of cardiovascular conditions, physical activity, and overall wellness [3, 4]. As these devices evolve toward continuous and autonomous operation, stable and sustainable power supply has emerged as one of the primary challenges limiting their long‐term usability [5, 6].

Despite rapid improvements in low‐power electronics, most wearable devices still rely on rechargeable batteries with limited size and energy capacity due to ergonomic and weight constraints [7]. In addition, frequent charging or battery replacement not only reduces user convenience but also restricts true all‐day or long‐term monitoring applications [8]. Consequently, self‐powered wearable systems based on energy harvesting technologies have attracted substantial attention as a promising alternative for sustainable operation [9]. Various approaches have been explored to harvest ambient energy sources, including thermal gradients [10], solar energy [11], and biomechanical motion [12]. Among these, human motion–driven energy harvesting is particularly attractive because biomechanical energy is continuously available during daily activities such as walking [13].

Previous studies have investigated piezoelectric [14], triboelectric [15], and electromagnetic energy harvesters [16] integrated into footwear or wearable platforms. However, most motion‐driven harvesters suffer from low energy output under low‐frequency and irregular human motion [17]. The intermittent nature of walking‐induced excitation often results in short‐duration energy generation, limiting overall power conversion efficiency. To overcome this limitation, mechanical strategies capable of transforming transient biomechanical inputs into sustained mechanical motion are required to enhance effective energy harvesting duration [18, 19]. A key limitation of existing biomechanical energy harvesters lies in the temporal mismatch between input and output: human motion provides impulsive, low‐frequency excitation, whereas wearable electronics require stable and continuous power. To address this fundamental challenge, strategies that decouple input intermittency from output continuity are required.

**FIGURE 1:**
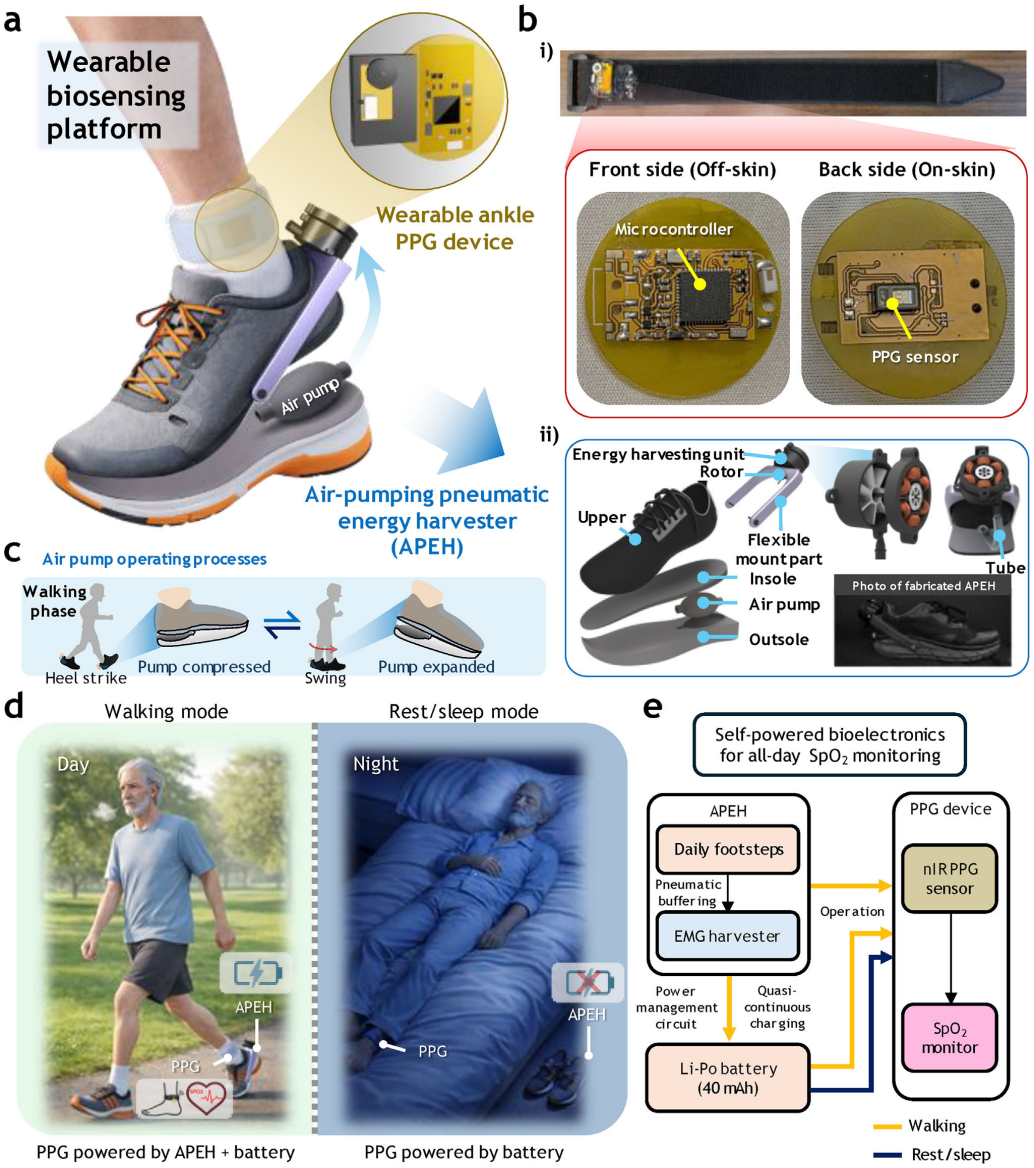
Overview of the self‐powered wearable bioelectronics integrating an air‐pumping pneumatic energy harvester and a wearable PPG sensor. (a) Schematic illustration of the wearable biosensing platform, showing the integration of the shoe‐mounted air‐pumping pneumatic energy harvester and the wearable ankle SpO_2_ PPG sensor. (b‐i) Photographs of the wearable PPG device, including front‐side (off‐skin) and back‐side (on‐skin) views with the microcontroller and PPG sensor, (ii) Schematic illustration and photograph of a wearable air‐pumping pneumatic energy harvester. (c) Working principle of the air‐pump‐based energy harvester during walking, illustrating cyclic compression and expansion of the pump. (d) Schematic illustration of walking and rest/sleep modes of the self‐powered wearable biosensing platform. During walking, the APEH harvests biomechanical energy from daily footsteps and transfers the generated electrical energy to the onboard battery, supplementing the stored battery energy for longer‐term use. The ankle‐mounted PPG module enables continuous SpO_2_ monitoring during both walking and rest/sleep, powered by the onboard battery. (e) System‐level diagram of the self‐powered wearable biosensing platform for all‐day SpO_2_ monitoring, showing energy harvesting, power management, battery charging/storage, and physiological signal monitoring.

In this work, we introduce a pneumatic energy buffering mechanism that converts transient mechanical inputs into sustained rotational motion, thereby extending the effective energy harvesting duration and enabling quasi‐continuous electrical output from inherently discontinuous human motion. Here, we report a self‐powered integrated wearable biosensing platform powered by daily footsteps for real‐time peripheral oxygen saturation (SpO_2_) monitoring as shown in Figure 1a. In the context of this work, the term “self‐powered” refers to the capability of the integrated wearable system to harvest electrical energy from the wearer's daily footsteps and internally deliver the harvested energy to the onboard rechargeable Li‐Po battery through the power‐management circuit, rather than complete battery‐free operation under all usage conditions. The proposed platform integrates a wearable ankle PPG device and an air‐pumping pneumatic energy harvester (APEH) to enable all‐day physiological monitoring (Figure 1b). Especially, a Velcro‐based fixation design of the wearable ankle PPG device secures the device firmly to the ankle, enabling stable biosignal acquisition with minimal motion‐induced noise even during walking. In addition, the proposed APEH employing an air‐pumping pneumatic mechanism converts body‐weight–induced impact energy into quasi‐continuous electrical energy during walking without noticeably disturbing natural gait (Figure 1c); the air pump embedded in the shoe passively undergoes cyclic compression and recovery, converting intermittent foot–ground interactions into quasi‐continuous rotation. Then, the mechanical rotary motion is easily converted into electrical energy using an electromagnetic generator (EMG), allowing extended energy harvesting from intermittent biomechanical excitation. To utilize the harvested energy for practical uses, a power management system integrating a boost‐type AC/DC converter and battery charging circuit is developed to directly charge a lithium‐polymer (LiPo) battery. Figure 1d,e schematically illustrates the operating concept of the all‐in‐one wearable bioelectronics and depicts the system‐level architecture showing the energy flow from daily activity to EMG‐based energy harvesting, battery charging/storage, and battery‐powered PPG‐based SpO_2_ monitoring, respectively. During walking, the APEH harvests biomechanical energy from daily footsteps and charges the onboard battery through the power‐management circuit, thereby supplementing the stored battery energy for longer‐term use. During rest or sleep, when gait‐energy input is unavailable, the PPG module continues monitoring using the stored battery energy. By linking pneumatic energy buffering, energy harvesting, and onboard battery charging, this work advances wearable biosensing platforms from fully battery‐dependent operation toward self‐powered architectures that can supplement stored energy using daily human motion.

## 2 Results and Discussion

### 2.1 Air‐Pumping Pneumatic Energy Buffering Harvester (APEH)

The proposed APEH operates as a mechanical energy buffer that temporally redistributes biomechanical input energy. During foot–ground interaction, compressive force generates pressurized airflow within the pneumatic chamber, which is subsequently released through a nozzle to drive rotor motion. Unlike direct‐contact energy harvesters, this process introduces a delay between input and output, resulting in sustained rotational motion after the initial excitation. This mechanism effectively transforms a low‐frequency impulsive input (∼1 Hz) into a higher‐frequency quasi‐continuous output, extending the energy generation window beyond the duration of foot contact. The persistence of rotational motion enables continuous electromagnetic induction, improving energy conversion efficiency compared to conventional transient harvesting approaches.

The air pump, a key component of the proposed APEH, is seamlessly embedded between a concave outsole and insole of a shoe, enabling direct conversion from plantar compression into rotational motion during walking. This design allowes the air pump to be compressed by body weight during stance while maintaining a relatively flat insole surface after compression, thereby reducing local protrusion. Unlike conventional wearable energy harvesters that impose additional mechanical resistance or require active user input, this structure passively utilizes the natural gait cycle, thereby minimizing additional user effort [20]. Plantar pressure distributions during walking were analyzed using a smart insole (Figure S1) to characterize the inherent biomechanical loading at the foot–ground interface. The measured pressure distribution by the smart insole clearly showed the typical gait sequence from heel strike to foot flat and toe‐off, revealing the spatiotemporal characteristics of plantar pressure during the gait cycle [21].

**FIGURE 2:**
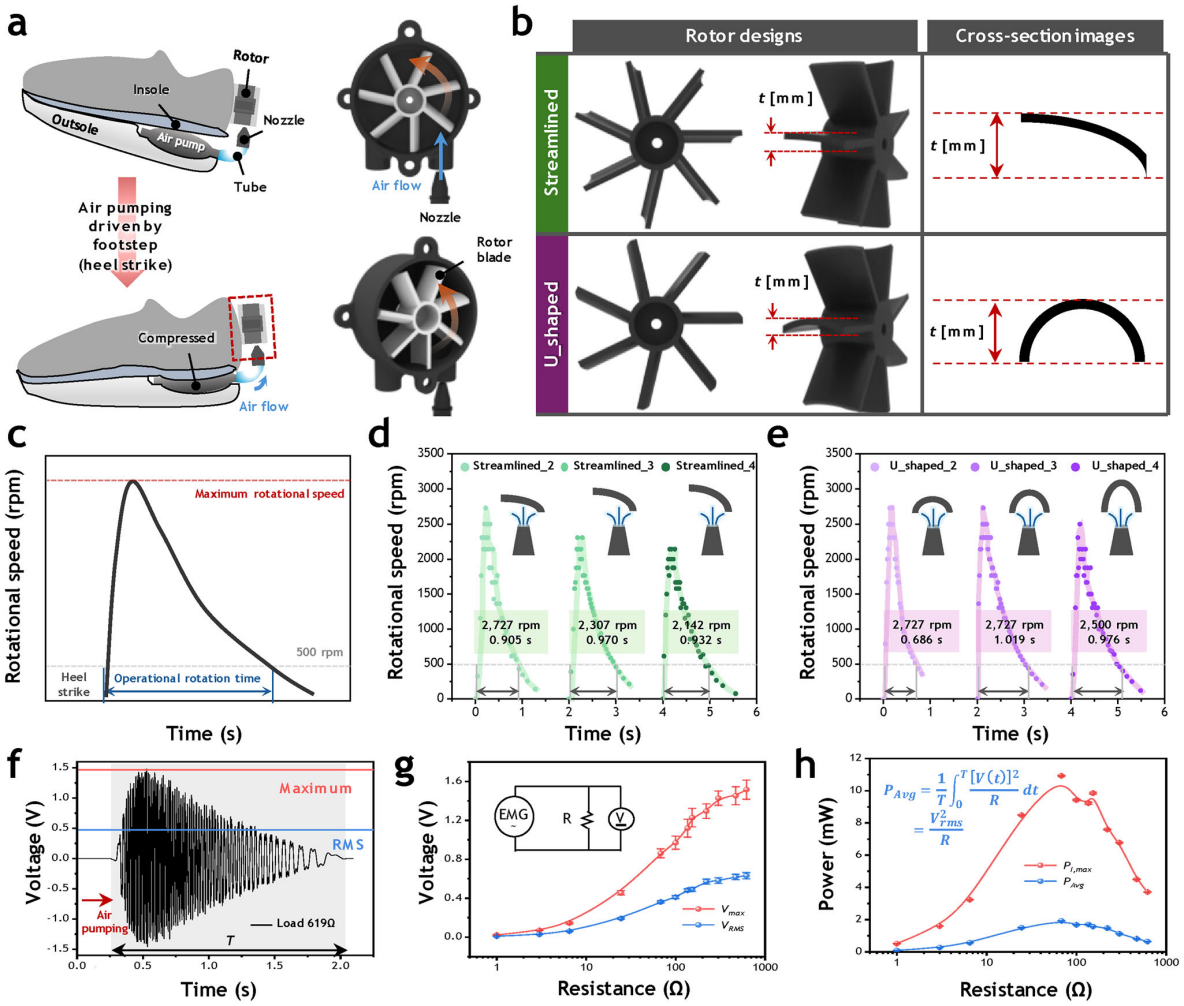
System analysis and performance evaluation of an air‐pumping pneumatic wearable energy harvester. (a) Schematic illustration of the air‐pumping mechanism integrated into the shoe, showing footstep‐induced compression and the resulting airflow through the nozzle, which drives rotational motion. (b) Schematic diagram of rotor designs (streamlined and U‐shaped) suggested in this study, and cross‐sectional diagrams of rotor blades. (c) Rotational speed profile during a single footstep, indicating the maximum rotational speed and duration for which the rotational speed exceeds 500 rpm for comparative analysis. (d, e) Rotational speed profile comparison of various rotor designs. (f) Representative output voltage signal under a load of 619 Ω, highlighting the maximum and RMS values. (g) Maximum (*V_max_*) and RMS values (*V_RMS_*) of output voltages as a function of load resistance to determine the maximum and time‐averaged power transferred from the EMG, respectively. (h) Maximum instantaneous power (*P_I, max_*) and time‐averaged power (*P_Avg_*) as a function of load resistance.

The rotational performance of the proposed APEH was first systematically evaluated. When the air pump is compressed under body weight during walking, pressurized airflow is generated and directed toward the rotor through a nozzle, as illustrated in Figure 2a. Because the proposed system relies on pneumatic energy transfer, the geometric configuration of both the nozzle and the rotor plays a critical role in determining overall energy conversion efficiency. To isolate the effect of rotor geometry, the nozzle configuration was kept constant while varying the blade designs; the effects of the nozzle configuration are discussed later in this section. In this study, three representative blade cross sections—plain, streamlined, and U‐shaped—were investigated. The rotational speeds for different blade shapes and parameters were compared under identical air‐pumping conditions, as shown in Figure 2b. For comparison, rotational speeds were measured for different rotor geometries, and both the maximum rotational speed and the duration over which the speed exceeded 500 rpm were extracted and presented together to quantitatively evaluate performance (Figure 2c).

As a control group, the plain rotor with a rectangular cross section exhibited a maximum rotational speed of approximately 1000 rpm (Figure S2a–b), while both streamlined and U‐shaped rotors achieved peak speeds exceeding 2500 rpm under the same conditions (Figures 2d–e). Given that the output of the energy harvester is proportional to the rotational speed, these results clearly demonstrate that the pneumatic energy harvesting performance strongly depends on the rotor geometry. To further optimize the rotational speed, the geometric parameters of the rotors were systematically adjusted. In the case of the streamlined design, the peak rotational speed tended to decrease as the blade curvature increased. With increasing curvature, a greater fraction of the nozzle‐ejected airflow is dissipated instead of being effectively converted into the driving force of the rotor. In contrast, the U‐shaped rotor exhibited the highest rotational speed and the longest rotation duration at a parameter value of 3 mm. Accordingly, this configuration was identified as the optimal condition for maximizing rotational performance.

Following the determination of the rotor structure, an electromagnetic generator (EMG) was designed and fabricated to convert the rotational motion into electrical energy (Figure S3). The EMG consists of an external stator with copper coils and an internal rotor embedded with six permanent magnets. To maximize energy conversion efficiency without significantly increasing the rotational inertia, the internal magnet configuration was considered. The performance of the EMG was first validated by externally driving the rotor using a motor as shown in Figure S4, confirming that the output voltage increases linearly with rotational speed over a wide frequency range. The EMG was then integrated with the air‐pump‐driven rotor to evaluate energy harvesting performance during walking. As shown in Figure 2f, the system produced a peak output voltage of approximately 1.5 V and an RMS voltage of approximately 0.5 V. Although the addition of the EMG slightly reduced the peak rotational speed due to increased inertia, it significantly extended the rotational duration, resulting in a sustained electrical output lasting longer than 1.5 s.

Energy harvesting experiments were conducted under a typical walking condition of 3.5 km/h for an average adult. During the gait cycle, the pressure between the heel and the ground increases from the onset of heel strike to the late flatfoot phase, within which the air pump is fully compressed [22]. Consequently, the local peak points in the harvester's output voltage profile can be plotted as voltage peak values proportional to the rotational speed (Figure S5). To further clarify the pneumatic energy‐buffering mechanism of the APEH, we evaluated the effect of air‐pump insertion on the transient heel‐strike pressure during walking. As shown in Figure S6, the heel‐region pressure response was compared with and without the air pump. The inserted air pump redistributed the heel‐strike load and delayed the time required to reach the peak pressure, indicating that the transient impact was temporarily buffered through bladder deformation and air compression before being released as airflow.

Based on this observation, we introduced a simplified physical model for the pneumatic energy‐buffering process, as illustrated in Figure S7. The model identifies the key parameters affecting APEH performance, including pressure–volume work, nozzle effect, rotor torque, moment of inertia, combined losses, and electromagnetic damping. Since the proposed pneumatic energy buffering mechanism is mainly composed of lightweight elements such as a compliant air bladder and airflow channels, it offers a practical advantage for integration into wearable systems as compared to conventional rotational motion converters. The detailed model description is provided in the Supporting Information. To experimentally support the role of nozzle geometry in the dynamic pneumatic response of the APEH, we further examined the effect of nozzle length on the output performance of the APEH while maintaining the same area ratio. The nozzle length influences the airflow resistance during air pump compression, which affects the dynamic deformation response and the subsequent transfer of pneumatic energy to the rotor [23]. By varying the nozzle length under identical input conditions, the time difference between the initial rotation onset (*t_i_*) and the time to reach maximum rotational speed (*t_f_*) was observed, indicating that the nozzle length significantly influences the transient energy transfer characteristics. The corresponding voltage peak values with different nozzle lengths are presented in Figure S8. The nozzle‐length‐dependent results demonstrate the APEH performance is affected by the dynamic pressure and airflow behavior determined by the nozzle geometry. Considering both performance and system‐level constraints such as device miniaturization, a nozzle length of 15 mm was selected as an optimal design, as it provides efficient energy transfer while maintaining a compact form factor with minimal loss. Furthermore, to assess potential energy losses within the pneumatic pathway, the effect of tube curvature between the air pump and the nozzle inlet was investigated as well. The results show that variations in the curvature radius have a negligible impact on the output performance (Figure S9), suggesting that the system is relatively insensitive to moderate geometric variations in the connecting tube.

To quantify the power output, both peak and RMS voltages were measured across a range of load resistances (Figure 2g), and the corresponding power delivered to the load was calculated. The peak voltage was used to determine the instantaneous maximum power (*P_I, max_*), while the RMS voltage was used to calculate the average power (*P_Avg_*) (Figure 2h). In addition, short‐circuit current measurements revealed current waveforms consistent with the voltage signals, with a maximum output current of approximately 20 mA, as shown in Figure S10. The maximum instantaneous standalone output power and time‐average power from the APEH were measured to be 10.9 and 1.93 mW, respectively. The energy harvested per step (regarding the average power of 1.93 mW) is estimated to be approximately 2–3 mJ corresponding to a conversion efficiency on the order of 10^−4^–10^−3^ relative to available biomechanical energy during walking. While comparable to state‐of‐the‐art wearable harvesters, the proposed system uniquely extends the duration of energy generation, enabling quasi‐continuous power output. To evaluate the effects of gait characteristics and individual variability on APEH performance, we further measured the RMS output voltage under different walking speeds and with subjects of different body weights. The results show the APEH output gradually increased as the walking speed increased, indicating that more frequent air pumping promotes higher rotor speed in the EMG (Figure S11). In addition, the output was not simply proportional to body weight in the initial measurements; however, subjects with different body weights exhibited comparable outputs after training with a simple APEH walking protocol (Figure S12). These results indicate that reliable APEH performance is primarily influenced by effective air‐bladder compression/reinflation within the gait cycle rather than body weight alone. Detailed experimental results and discussion are provided in the Supporting Information.

In addition, the feasibility of the proposed harvester was demonstrated during treadmill walking at a speed of 3.5 km/h (Video S1), confirming the effectiveness of the proposed system under realistic operating conditions. Importantly, the extended rotation duration increases the effective duty cycle of energy generation, which is a critical factor for enabling continuous operation of wearable electronics despite similar absolute efficiency. Although the absolute conversion efficiency is comparable to existing systems, the key advantage lies in the increased effective duty cycle of energy generation, which directly enables continuous operation of wearable electronics. Compared to triboelectric and piezoelectric shoe‐mounted energy harvesters, which typically produce short‐duration, impulsive outputs, the proposed pneumatic system introduces a buffering mechanism that enables sustained energy generation. This distinction is critical for powering continuous wearable electronics, where stable energy supply is required. To examine whether the APEH‐integrated shoe noticeably affects gait, we compared walking with a normal shoe and with the APEH‐integrated shoe using synchronized walking video and smart‐insole plantar‐pressure visualization (Video S2). The plantar‐pressure distribution was provided as a color map by the smart‐insole application; therefore, the result was used as a qualitative gait assessment and no apparent change in the overall walking pattern or plantar‐pressure distribution was observed under the tested condition, suggesting that the in‐shoe air‐pump structure does not noticeably disturb natural walking.

### 2.2 Fabrication and Validation of the Wearable Ankle PPG Device

**FIGURE 3:**
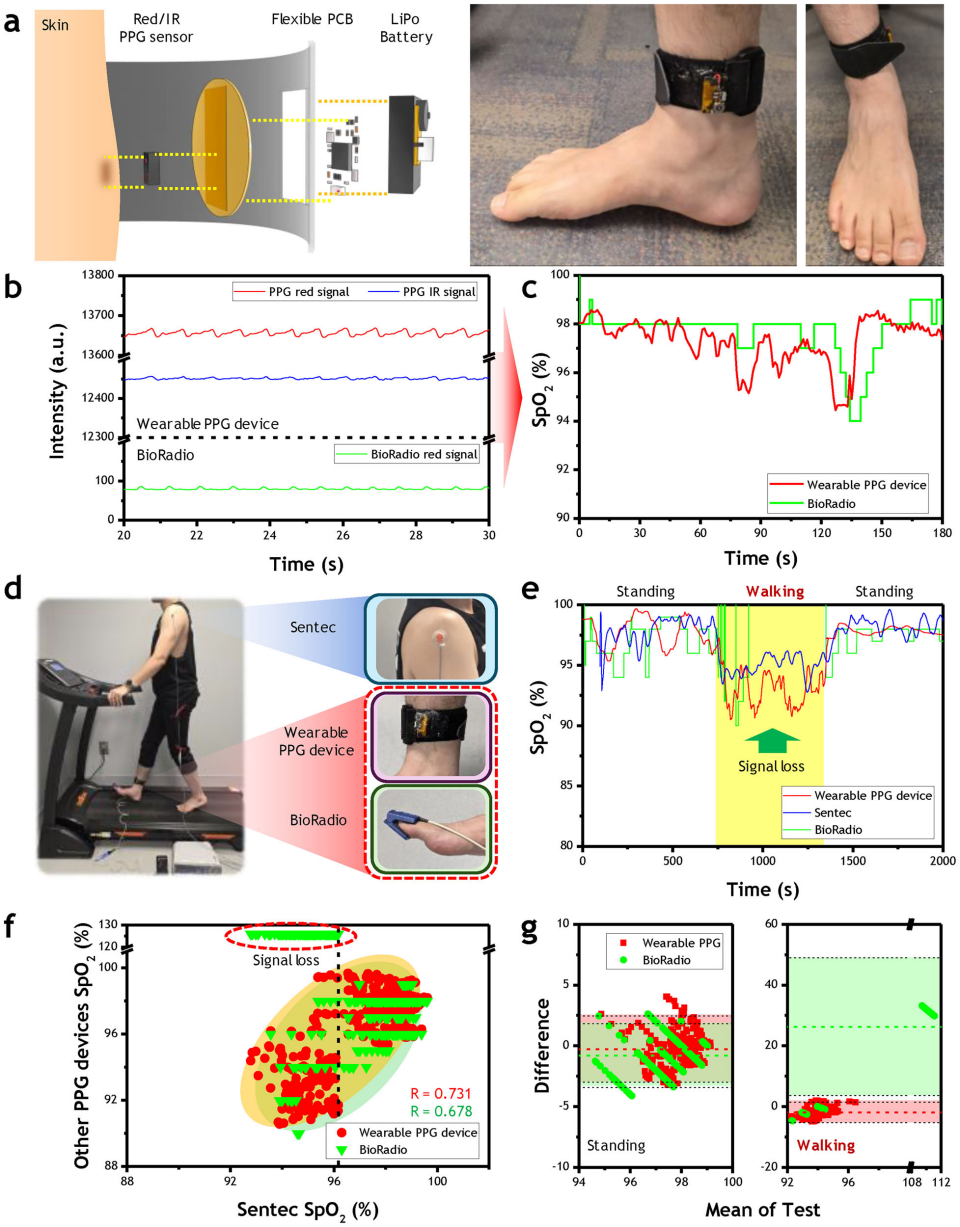
Characterization and validation of the wearable ankle PPG device. (a) Schematic illustration of the wearable ankle PPG device and photograph of a subject wearing the device. (b) Red and infrared PPG signals of the wearable ankle PPG device and red PPG signal of the reference BioRadio. (c) Calculated SpO_2_ of the wearable ankle PPG device and the BioRadio. (d) Illustration of the SpO_2_ test setup with the wearable ankle PPG device and commercial devices (BioRadio and Sentec SDM). (e) SpO_2_ results from the wearable ankle PPG device and commercial devices while standing and walking. (f) Correlation analysis of the Sentec SDM SpO_2_ data and others (the wearable ankle PPG device and the BioRadio). (g) Bland–Altman analysis of the Sentec SDM SpO_2_ data, the wearable ankle PPG device, and the BioRadio. Data were pooled from all 3 subjects and subsampled for visualization purposes; all statistical analyses were conducted on the complete dataset.

To demonstrate the practical applicability of the shoe‐mounted APEH, all‐day lower‐limb SpO_2_ monitoring was selected as a target application for daily healthcare [24, 25, 26]. Since the proposed APEH is integrated within the shoe, a biosensor capable of monitoring in close proximity to the foot was required. Ankle‐based PPG monitoring has been previously demonstrated as a feasible measurement site for physiological signal acquisition [27, 28]. Therefore, a wearable PPG device for ankle‐mounted SpO_2_ monitoring was designed and fabricated. As shown in Figure 3a, the device integrates MAX30102, which measures optical PPG sensing with red (660 nm) and infrared (880 nm) LEDs on a flexible printed circuit board (fPCB), thereby enabling conformal interfacing with the lower‐leg anatomy (Figure S13). The platform was powered by a 40 mAh LiPo battery to support wearable operation. The assembled device was secured to an adjustable strap to allow use across subjects with different calf circumferences. To assess the feasibility of the wearable ankle PPG device, three subjects were enrolled, and its performance was compared with that of a BioRadio system used as a reference standard. Since the BioRadio PPG sensor is clip‐based, it was attached to the hallux, and measurements were performed while the foot remained stationary (Table S1). Figure 3b presents the red and infrared signals from a subject acquired with the wearable ankle PPG device, along with the red‐channel signal obtained from the BioRadio system. In both devices, pulse waveforms corresponding to cardiac activity were clearly observed. To further confirm signal reliability, pulse rate was extracted from the raw PPG signals of both devices (Figure S14a), yielding a Pearson correlation coefficient of 0.978, indicating that both devices consistently captured the underlying cardiac pulsatile signal (Figure S14b). Based on these signals, SpO_2_ was subsequently calculated (Figure 3c). SpO_2_ was calculated using the ratio‐of‐ratios (RoR) method with the equation;

$$\mathrm{Sp}O_{2}=116.91\;-\;37.663\;\times \;z$$

$$z=\frac{\left[IR\left(ac\right)/IR\left(dc\right)\right]}{\left[Red\left(ac\right)/Red\left(dc\right)\right]}$$

where, *IR(ac)* and *Red(ac)* represent the pulsatile (alternating) components of the infrared and red PPG signals, respectively, corresponding to the arterial blood volume changes with each cardiac cycle, while *IR(dc)* and *Red(dc)* represent the non‐pulsatile (direct) baseline components, which reflect the optical properties of static tissue, venous blood, and other non‐pulsatile absorbers. The calibration coefficients (116.91 and 37.663) were adopted from the previous literature [29] where they are explicitly provided in Figure S15. To further confirm that both systems captured the same overall trend, subjects intentionally held their breath for 20 s starting at 100 s after the start of measurement to induce a reduction in SpO_2_. Although a temporal offset in the response was observed between the two devices, both systems exhibited a comparable decrease in SpO_2_. These results confirm that the wearable ankle PPG device can reliably detect lower‐limb PPG signals.

We then evaluated the wearable ankle PPG device during walking. For comparison of SpO_2_, a commercial SpO_2_ monitoring device (Sentec SDM) was attached to the shoulder, and a BioRadio clip‐based PPG sensor was placed on the hallux (Figure 3d) [30]. As shown in Figure 3e, during standing, all three devices exhibited similar trends in SpO_2_ variation, although differences in response delay were observed due to differences in device location and local blood perfusion characteristics. Changes in SpO_2_ were first detected by the Sentec SDM, followed by the wearable ankle PPG device and then the BioRadio system, suggesting that the response delay was primarily governed by the relative distance from the heart. However, when the subjects started walking, the BioRadio system placed on the hallux failed to acquire stable signals because pressure changes at the toe during gait interfered with signal detection, resulting in signal loss. In contrast, the wearable ankle PPG device continuously captured PPG‐related signal variations during walking. Although the measured SpO_2_ values were lower than those obtained from the Sentec SDM, which exhibited relatively stable signal output, the wearable ankle PPG device maintained continuous operation under dynamic conditions. After walking was terminated, the BioRadio system resumed normal operation, and all three devices returned to SpO_2_ values within the normal range. Also, pulse rate was extracted from the raw PPG signals of all devices and compared across conditions to further validate signal reliability beyond SpO_2_ (Figure S16a). The wearable ankle PPG device maintained a high pulse rate correlation with the Sentec SDM (r = 0.957) across standing and walking conditions. The BioRadio showed a reduced correlation (r = 0.771) across standing and walking conditions due to the 85.8% signal loss rate at the toe site during walking, which recovered to r = 0.976 when signal loss events were excluded (Figure S16b).

The SpO_2_ values obtained from the wearable ankle PPG device and the BioRadio system from three subjects were plotted against those measured by the shoulder‐mounted Sentec SDM, which served as the relatively stable reference under ambulatory conditions (Figure 3f). Data points were subsampled for visualization purposes; all statistical analyses were conducted on the complete dataset. Although both devices exhibited differences relative to the Sentec SDM measurements due to sensor placement and response delay, both showed an overall linear trend. However, during walking, the BioRadio system placed on the hallux frequently exhibited signal loss due to compression‐induced motion artifacts at the toe, resulting in artifact values of 127%, which correspond to out‐of‐range signal acquisition failure outputs generated when valid SpO_2_ cannot be computed at the toe measurement site. It should be noted that this signal loss is attributable to the toe measurement site rather than the device itself. In contrast, the wearable ankle PPG device produced comparable SpO_2_ values without such frequent signal‐loss artifacts. The Pearson correlation coefficient between the Sentec SDM and the wearable ankle PPG device was 0.731, while that between the Sentec SDM and the BioRadio was ‐0.804 when signal loss events were included, recovering to 0.678 after excluding signal loss events from the BioRadio data, further confirming that the signal loss at the toe site was the primary source of measurement distortion.

The agreement between the wearable ankle PPG device or the BioRadio system and the Sentec SDM reference was further evaluated using Bland–Altman analysis. In this analysis, the mean of the test and the difference were defined as

$$\mathrm{Mean}\;\mathrm{of}\;\mathrm{test}\;=\frac{X_{\mathrm{test}}+X_{\mathrm{ref}}}{2}\;$$

$$\mathrm{Difference}\;=X_{\mathrm{test}}\;-X_{\mathrm{ref}}$$

where *X*_test_ denotes the SpO_2_ value measured by the wearable ankle PPG device or the BioRadio system and *X*_ref_ denotes the SpO_2_ value measured by the Sentec SDM. Figure 3g shows the Bland–Altman plots obtained during standing and walking of three subjects. The center dashed line indicates the mean difference, and the shaded region represents the limits of agreement (LoA), calculated as the mean difference ± 1.96 standard deviations of the differences. During standing, the wearable ankle PPG device exhibited a mean difference of −0.273, with LoA ranging from −3.009 to 2.463 and 95% CI of LoA from −3.305 to −2.714 and from 2.168 to 2.758, which is close to zero compared with the BioRadio, which showed a mean difference of −0.802, LoA ranging from −3.435 to 1.830, and 95% CI of LoA from −3.719 to −3.150 and from 1.546 to 2.114, indicating closer agreement with the Sentec SDM reference. During walking, the BioRadio system placed on the hallux exhibited a mean difference of 26.290 due to signal loss events at the toe site, indicating that toe‐based PPG measurement is not suitable for reliable SpO_2_ monitoring under dynamic conditions. When signal loss events were excluded, the BioRadio system showed a mean difference of −1.969, with LoA ranging from −4.903 to 0.965 and 95% CI of LoA from −6.046 to −3.758 and from −0.179 to 2.109, comparable to that of the wearable ankle PPG device. In contrast, the wearable ankle PPG device maintained a mean difference of −1.915, with LoA ranging from −5.263 to 1.434 and 95% CI of LoA from −5.798 to −4.728 and from 0.899 to 1.969 during walking, remaining within a practically acceptable range. These results demonstrate that the wearable ankle PPG device can provide reliable lower‐limb SpO_2_ measurements even during ambulation.

The wearable ankle PPG device can also be used for disease monitoring based on raw ankle PPG signals [31, 32]. Figure S17 presents the results obtained according to a venous reflux protocol. These findings suggest that the wearable ankle PPG device has potential for use not only in daily SpO_2_ monitoring but also in at‐home diagnostic applications. Although the device was only shown to be feasible in this paper, further research will be conducted with the patient data collection. These findings provide a preliminary observation suggesting that ankle PPG signals may reflect venous hemodynamic changes. While this result is exploratory in nature, it indicates that ankle‐mounted PPG sensing may offer potential avenues for future investigation beyond SpO_2_ monitoring. Rigorous clinical validation with patient data would be required before any diagnostic application could be considered. Unlike conventional distal PPG sensing, which is highly susceptible to pressure‐induced artifacts during ambulation, the ankle‐based sensing location provides a mechanically stable interface with reduced local deformation, enabling robust signal acquisition under dynamic conditions. These results demonstrate that ankle‐based PPG sensing is inherently more suitable for ambulatory monitoring than conventional distal configurations, providing a robust platform for continuous biosignal acquisition under dynamic conditions.

### 2.3 Power Management System for Commercial Battery Charging

**FIGURE 4:**
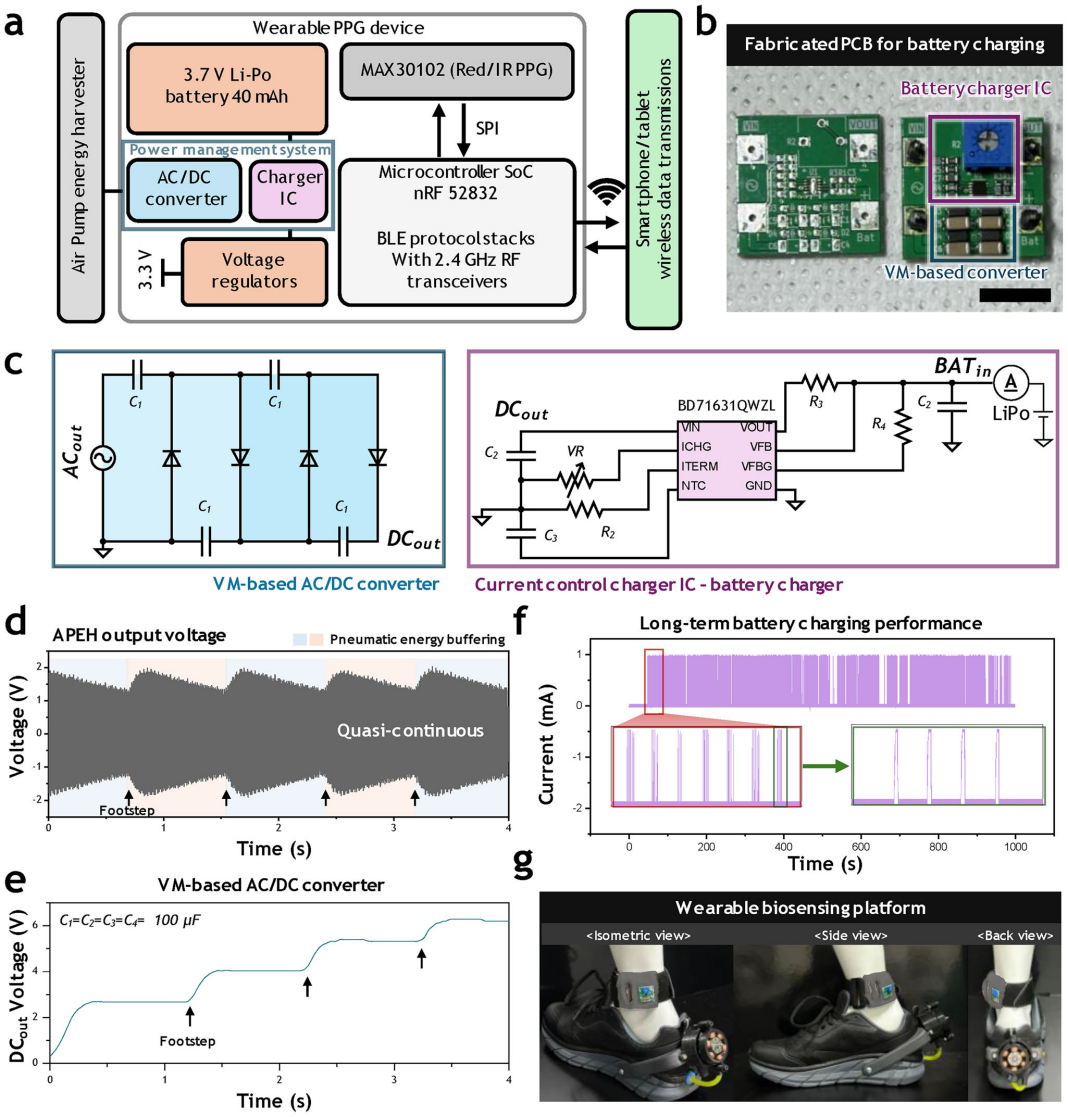
Power management circuit design and system integration for battery charging of the self‐powered wearable biosensing platform. (a) System‐level block diagram of the self‐powered wearable biosensing platform integrated with the air‐pump energy harvester, power management system including AC/DC conversion and current‐controlled charging, and PPG sensor. (b) Photograph of the fabricated printed circuit board (PCB) for battery charging, highlighting the voltage multiplier (VM)‐based AC/DC converter and charger IC. (c) Schematic circuit diagram of the VM‐based AC/DC converter (left) and the current‐control charger IC for battery charging (right). (d) AC output voltage of the air pumping pneumatic energy harvester (APEH) indicating the pneumatic energy buffering mechanism. (e) DC output voltage of the VM‐based AC/DC converter with the capacitance of 100 µF. (f) Long‐term current measurement from the charger IC to the lithium‐polymer (LiPo) battery, demonstrating stable battery charging and current‐controlled power management. (g) Photographs of the fully integrated wearable biosensing platform, including isometric, side, and back views.

To integrate the proposed air‐pump energy harvester with the wearable ankle PPG device toward a self‐powered biosensing platform, a power management system capable of charging a commercial LiPo battery was developed, and a schematic illustration of the fully integrated system is presented in Figure 4a. In this study, a commercially available 40 mAh LiPo battery was employed as the power source for the wearable ankle PPG device. For battery charging, the power management system consists of a voltage multiplier (VM)‐based AC/DC converter and a current‐controlled charger IC. The entire system was designed and fabricated on a printed circuit board (PCB), as shown in Figure 4b. Specifically, the output voltage of the energy harvester is insufficient to directly charge the LiPo battery, whose operating voltage is relatively higher. Therefore, a VM circuit, composed of multiple diodes and capacitors, was employed to simultaneously boost the voltage and rectify the AC output generated by the EMG, as illustrated in Figure 4c. Prior to evaluating the power‐management circuit, the time‐domain output voltage of the APEH during continuous walking was examined, as shown in Figure 4d. Owing to the sustained rotor motion after each footstep, the oscillatory voltage output persisted between successive steps, resulting in a quasi‐continuous AC waveform rather than isolated voltage pulses.

The performance of the VM‐based AC/DC converter was first evaluated using a function generator, and the effect of capacitance values was systematically investigated (Figure S18a–c). The working principle of the VM‐based converter is schematically described in Figure S18d,e. The VM‐based converter sequentially charges and stacks voltages across capacitors through alternating diode conduction during each AC cycle, resulting in stepwise voltage boosting [33]. This process simultaneously rectifies the AC input and generates a higher DC output suitable for battery charging. The capacitance value significantly influences the charging and discharging behavior of the VM circuit; a larger capacitance leads to slower charging but improved voltage retention with reduced ripple, whereas a smaller capacitance enables faster voltage rise but results in rapid discharge and larger fluctuations [34]. To clarify the power‐management behavior, we further characterized the battery‐charging current of the VM‐based AC/DC converter under different storage capacitances without a battery charger IC (Figure S19). In this study, a capacitance of 100 µF was selected as an optimal value to ensure stable output voltage, reliable power delivery, and small form factor. Direct battery‐charging tests showed that increasing the storage capacitance stabilized the transient charging current generated by repeated footsteps, although larger capacitors should be balanced against wearable form‐factor constraints. The DC output voltage of the selected VM‐based AC/DC converter was then monitored during repeated footsteps, as shown in Figure 4e. The rectified voltage increased stepwise with each footstep and was maintained between successive steps, confirming both voltage boosting and the accumulation of harvested energy. These results demonstrate that the converter provides a sustained DC output suitable for subsequent battery charging.

For efficient battery charging, a consistent current supply is essential; however, the output of the VM‐based AC/DC converter inherently exhibits transient and fluctuating characteristics due to the irregular nature of walking phases. To address this issue, a commercially available charger IC with current regulation capability, particularly suitable for low‐current operation, was incorporated to improve energy transfer efficiency and mitigate potential battery degradation. The block diagram of the charger IC, based on constant current/constant voltage (CC/CV) regulation, is illustrated in Figure S20a. The overall PCB layout of the power management system for battery charging based on the air‐pumping pneumatic energy harvester is shown in Figure S20b. In addition, controlled DC power‐supply tests confirmed that the 1 mA setting of the charger IC corresponds to the programmed current‐limiting threshold (Figure S21). To verify the stable battery charging capability of the proposed power management system, a long‐term charging experiment was conducted by monitoring the output current delivered to the LiPo battery, as shown in Figure 4f. The output current remained stable without noticeable decay during the test, suggesting that the pneumatic energy‐buffering mechanism can sustain battery charging without significant performance degradation under the long‐term operation. Unlike gear‐ or rack‐based mechanical transmission systems, which rely on direct solid–solid contact and may require component replacement due to frictional wear, the proposed APEH converts footstep‐induced compression into airflow‐driven rotor rotation through an air‐pumping structure without direct mechanical contact. In addition, the air pump is made of an elastomeric structure that undergoes reversible compression and passive reinflation during walking, thereby reducing the risk of plastic deformation under body‐weight loading. During the experiment, the charging current was set to 1 mA by adjusting a variable resistor in the charger IC to 500 kΩ. The results demonstrate that the fabricated PCB successfully converts high‐frequency AC signals generated from walking motion into a regulated DC current suitable for battery charging. Consequently, Figure 4g presents photographic images of the self‐powered, fully integrated biosensing platform comprising the APEH and wearable ankle PPG sensor.

### 2.4 All‐In‐One Integrated Biosensing Platform

**FIGURE 5:**
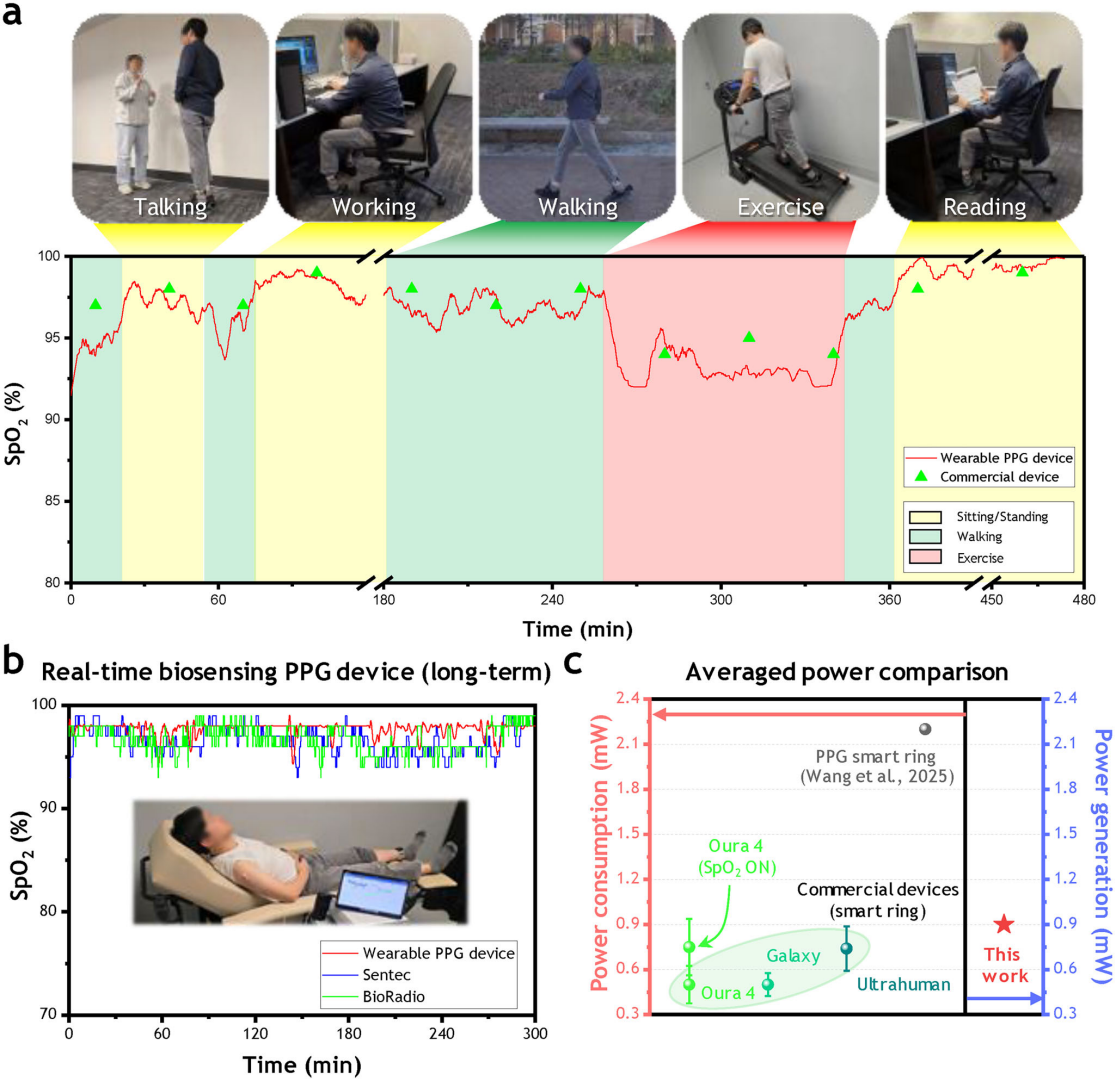
Demonstration of the energy harvester‐integrated wearable ankle PPG platform in daily life. (a) Photos showing an example of a subject wearing the energy harvester‐integrated wearable ankle PPG platform and resulting SpO_2_ changes in a daily routine. (b) Sleep monitoring of the energy harvester‐integrated wearable ankle PPG platform compared with commercial devices. (c) Comparison of generated power with the power consumption of wearable electronics to assess the feasibility of the self‐powered system.

We finally demonstrated the self‐powered integrated wearable biosensing platform during daily activity; a subject first wore the platform and underwent continuous measurement for 8 h during daily activities. The upper panel of Figure 5a shows representative photographs acquired under each condition. During the measurement period, the subject performed a range of routine activities, including standing or sitting while working, reading, or conversing, walking during transitions between locations, and exercise, while SpO_2_ was continuously monitored. To minimize interference with the subject's activity, the reference BioRadio system was applied to the index finger for 20 s at 30 min intervals to obtain intermittent SpO_2_ measurements. The ankle‐mounted device did not interfere with daily activity and enabled continuous monitoring without signal loss across diverse behavioral conditions. Moreover, the SpO_2_ values obtained at each time point showed trends highly comparable to those measured by the BioRadio reference. In addition to SpO_2_, heart rate was continuously extracted from the raw PPG signals throughout the 8 h monitoring period, revealing activity‐dependent HR variations consistent with the physiological demands of each condition (Figure S22). These results demonstrate that the energy harvester‐integrated wearable ankle PPG device enables long‐term SpO_2_ monitoring through PPG analysis without causing discomfort during routine daily use. In addition, the compact built‐in battery enables SpO_2_ monitoring even under conditions in which energy harvesting is not available, such as during sleep. For nighttime evaluation, the subject slept while wearing the Sentec SDM, BioRadio, and wearable ankle PPG device on the shoulder, finger, and ankle, respectively (Figure S23). As shown in Figure 5b, the SpO_2_ profiles measured over 6 h of sleep by the wearable ankle PPG device, Sentec SDM, and BioRadio exhibited similar overall trends. The mean SpO_2_ values measured by the wearable ankle PPG device, Sentec SDM, and BioRadio were 96.75 ± 1.33%, 97.40 ± 2.49%, and 96.80 ± 1.22%, respectively, confirming comparable SpO_2_ tracking across all three devices during sleep. Similarly, the mean pulse rate values were 62.92 ± 1.76, 63.71 ± 2.46, and 64.34 ± 2.44 bpm, respectively, demonstrating consistent cardiac pulsatile signal capture across all devices throughout the sleep monitoring period. (Figure S24) The SpO_2_ signal from the ankle PPG while sleeping suggests potential for future investigation into sleep‐related applications, including sleep‐disordered breathing detection, though further studies with appropriate reference systems are needed [35]. These results confirm that the developed platform enables continuous monitoring not only during daily activity but also during sleep, thereby supporting all‐day SpO_2_ measurement, with continuous power support from the wearable energy harvester. Also, the improved robustness originates from reduced local deformation and pressure variation at the ankle compared to distal sensing sites, which are strongly affected by gait‐induced contact forces.

Following system integration, the electrical power delivered to the battery was quantified based on the previously measured current–time profiles (Figure 4d), yielding a time‐averaged power of 0.9 mW under realistic operating conditions. To place the battery‐delivered power in a broader performance context, Table S2 presents a quantitative comparison based on practical output metrics and dimensions of integrated systems with previously reported shoe‐mounted wearable energy harvesters [19, 36, 37, 38, 39]. This comparison highlights that the proposed APEH benefits from pneumatic energy buffering, which extends the energy conversion process and thereby provides a relatively high time‐averaged output power compared with conventional shoe‐mounted harvesters that typically generate intermittent electrical outputs. In addition, the delivered power after power management confirms that a substantial fraction of the harvested energy can be transferred to the storage element, supporting the practical system‐level integration of the harvester with wearable electronics. To further assess the practical relevance of this power level, the power consumption of several commercially available smart sensors and a wearable sensor reported in the literature was compiled and compared, as shown in Figure 5c. The power consumption of commercial smart sensors (e.g., smart rings) typically ranges from 0.4 mW to 1.0 mW depending on operating conditions, with an additional increase of approximately 0.2–0.3 mW when SpO_2_ measurement is activated. In comparison, a ring‐type wearable PPG SpO_2_ sensor reported in the literature exhibits an average power consumption of approximately 2.2 mW [40]. These results indicate that the transferred power from the wearable energy harvester is already within the operational range of low‐power wearable devices and can be further improved through system‐level optimization. Such improvements could enable a fully self‐powered health monitoring platform capable of continuous physiological signal acquisition without external power sources. This work highlights the feasibility of coupling biomechanical energy harvesting with wearable biosensing systems, paving the way for sustainable and autonomous health monitoring technologies.

## 3 Conclusion

In this work, we introduce pneumatic energy buffering as a mechanism to decouple intermittent biomechanical input from continuous electrical output and report a self‐powered wearable biosensing platform that integrates an APEH with the wearable ankle PPG sensor, which enables continuous physiological monitoring. By leveraging biomechanical energy from daily activities, the proposed pneumatic energy buffering system enables sustained power generation and efficient energy management through a VM‐based AC/DC converter and current‐controlled charging circuit. Notably, the air‐pumping pneumatic rotary converter features a lightweight design that minimizes additional user effort during natural human motion. Furthermore, by effectively transforming intermittent and irregular footstep inputs into sustained rotational motion, the system enables continuous and high‐efficiency energy generation. The optimized pneumatic–electromagnetic conversion mechanism achieves stable power delivery, reaching a time‐averaged output of 0.9 mW, which is within the operational range of low‐power wearable devices. Through systematic device‐level optimization, circuit design, and system‐level integration, the platform demonstrates reliable operation under realistic conditions, enabling extended‐duration biosignal acquisition with reduced reliance on external power sources. Moreover, the wearable ankle PPG device, secured via a Velcro‐based design, provides robust and stable signal acquisition with minimal signal loss even under motion‐induced interferences. This work shifts the paradigm of wearable energy harvesting from instantaneous energy extraction to temporally buffered energy conversion, enabling continuous operation of wearable bioelectronics under realistic human motion conditions. While the current study demonstrates the feasibility of an energy‐harvesting‐integrated ankle PPG platform for continuous SpO_2_ monitoring, further studies with larger and more diverse subject cohorts, including subjects with varying skin tones across the Fitzpatrick scale and different shoe sizes, broader physiological conditions, and clinical validation in collaboration, are needed to fully establish its generalizability and utility as a reliable healthcare monitoring tool. In addition, further miniaturization and lightweight design of the APEH‐integrated shoe will be required to improve user comfort during wearable operation. Nevertheless, the findings of this study are expected to provide a foundational framework for the development of harvesting‐assisted biosensing platforms, particularly for applications requiring long‐term, continuous physiological monitoring in everyday environments.

## 4 Experimental Section

### 4.1 Fabrication and Integration of a Wearable Energy Harvester and Power Management

The mechanical components of the APEH and wearable housing were fabricated using a stereolithography (SLA) 3D printing process (Form 2, Formlabs). Structural parts, including the enclosure and internal supporting components, were designed to accommodate rotational motion while maintaining compact wearable form factors. Mechanical elements required for the rotational energy harvesting module, including shafts and permanent magnets, were commercially procured from MISUMI Korea. Neodymium permanent magnets with dimensions of 10 × 5 × 5 mm^3^ were employed to generate magnetic flux variation during rotation. The electromagnetic generator was constructed using a copper coil with 300 turns to achieve efficient electromechanical energy conversion.

For electrical energy management, a dedicated power management system was implemented to regulate harvested energy and enable stable device operation. A battery charging integrated circuit (BD71631QWZ, ROHM Co., Ltd.) was adopted for current control. The printed circuit board (PCB) fabrication and component mounting were outsourced to NTREX Co., Ltd. The integrated system combines mechanical energy harvesting, energy storage, and power conditioning modules to support continuous operation of the wearable biosensing platform.

### 4.2 Characterization of Air‐Pumping Pneumatic Energy Harvester (APEH)

To measure the output voltage of the proposed energy harvester, a digital phosphor oscilloscope (DPO2002B, Tektronix), a high‐voltage probe with an input impedance of 40 MΩ (P5100A, Tektronix), and a pre‐current amplifier (SR570, Stanford Research Systems) were used to measure the output voltage and current. A system electrometer (Model 6514, Keithley Instrument) was used to measure the open‐circuit voltage and short‐circuit current.

A smart insole system (Balance, Salted) was employed to measure plantar pressure distribution during walking and to verify the preservation of a natural gait pattern. The rotational speed of the air‐driven rotor was quantitatively analyzed using a high‐speed camera (FASTCAM MINI‐UX50, Photron Inc.). Cyclic compression tests for calibration of a commercially available pressure sensor were performed using a universal testing machine (AGS‐X, Shimadzu). To evaluate the operating performance of the proposed AC/DC converter, a dual‐channel arbitrary function generator (AFG3022, Tektronix) was utilized. No apparent deviation in gait sequence, pressure distribution, or temporal characteristics was observed compared to normal walking conditions.

### 4.3 Fabrication of the Wearable Ankle PPG Device

The fPCB for the wearable ankle PPG device was designed in Altium. The circuit radius is 2.5 cm, and the actual board dimension is 2.2 cm × 1.1 cm; components were placed on both the top and bottom to achieve a small, compact design. The designed circuit board was manufactured by PCBway. The fPCB was soldered in the laboratory with conductive solder paint and tacky flux under 180°C. The total electronic device weight of the on‐body electronics was 15 g, including a 40 mAh LiPo battery and wiring.

### 4.4 Characterization of the Wearable Ankle PPG Device

The performance of the wearable ankle PPG device was evaluated using a BioRadio system (Great Lakes NeuroTechnologies) and a Sentec Digital Monitor (SDM; Sentec) as reference devices. The wearable ankle PPG device was mounted on the ankle, the BioRadio sensor was attached to the hallux, and the Sentec SDM was placed on the shoulder. The subject then underwent a treadmill protocol consisting of 10 min standing, 10 min walking, and 10 min standing. SpO_2_ signals obtained from the three devices were recorded and compared throughout the test.

### 4.5 SpO_2_ Calculation Algorithm for the Wearable Ankle PPG Device

For SpO_2_ calculation from the wearable ankle PPG device, red (660 nm) and infrared (880 nm) PPG signals were acquired using the MAX30102 sensor. Ambient light components were first rejected from both channels. The pulsatile AC component was then extracted using a band‐pass filter centered at the heart rate with a bandwidth of ± 0.5 Hz, while the DC component was obtained using a low‐pass filter with a cutoff frequency of 0.1 Hz. To reduce motion‐related and irregular pulse artifacts, abnormal beats were excluded based on template‐distance beat rejection. The remaining valid beats were used to calculate the average beat amplitude within a 7 s sliding window. The ratio‐of‐ratios parameter, *z*, was then calculated as

$$z=\frac{IR\left(ac\right)/IR\left(dc\right)}{Red\left(ac\right)/Red\left(dc\right)}$$

where *IR(ac)* and *Red(ac)* represent the AC amplitudes of the infrared and red PPG signals, respectively, and *IR(dc)* and *Red(dc)* denote the corresponding DC components. SpO_2_ was then estimated using the linear calibration equation.

### 4.6 Demonstration of Self‐Powered Wearable Biosensing Platform

For daytime evaluation, the subject wore the wearable biosensing platform for 8 h during routine daily activity. Reference measurements were obtained using the BioRadio system, which was intermittently attached to the index finger for 20 s at 30 min intervals. For nighttime evaluation, the subject wore the wearable biosensing platform, BioRadio, and Sentec SDM during sleep for 6 h. SpO_2_ signals obtained from each device were recorded and compared.

### 4.7 Human Subject Study

All experiments involving human participants were reviewed and approved by the Institutional Review Board of the Georgia Institute of Technology (IRB No. IRB2026‐60). All procedures were conducted in accordance with the approved protocol and relevant institutional guidelines. In accordance with ethical guidelines, all participants provided written informed consent prior to the study. They were given a detailed explanation of the experimental protocol, potential risks, and their right to withdraw at any time.

## Author Contributions

**Ji‐Seok Kim**: conceptualization, writing – original draft, writing – review and editing, investigation, methodology, validation, formal analysis, data curation. **Tae Woog Kang**: investigation, methodology, conceptualization, validation, writing – original draft, software. **Hyunjoon Yoo**: investigation, validation, formal analysis. **Jawon Ha**: formal analysis, validation, visualization. **Yunuo Huang**: visualization. **Hee Han**: project administration, investigation. **Chi Won Ahn**: investigation, project administration. **Woon‐Hong Yeo**: funding acquisition, writing – review and editing, conceptualization, validation, supervision, methodology. **Il‐Kwon Oh**: writing – review and editing, supervision, conceptualization, funding acquisition, investigation, methodology, project administration, visualization.

## Conflicts of Interest

The authors declare no conflicts of interest.

## Supporting information




**Supporting File 1**: advs77158‐sup‐0001‐SuppMat.docx.

**Supporting File 2**: advs77158‐sup‐0002‐VideoS1.mp4.

**Supporting File 3**: advs77158‐sup‐0003‐VideoS2.mp4.

## Data Availability

The data that support the findings of this study are available from the corresponding author upon reasonable request.
