# Supplementary material for: Self‐Powered All‐in‐One Wearable Bioelectronics Driven by Pneumatic Energy Buffering of Daily Footsteps
Source: Adv Sci (Weinh). 2026 Aug 11;13(61):e77158. doi: 10.1002/advs.77158 (PMC13460423; doi:10.1002/advs.77158)
Supplement: Supplementary file 1 [file ADVS-13-e77158-s001.docx]

Supporting information

Self-Powered All-in-One Wearable Bioelectronics Driven by Pneumatic Energy Buffering of Daily Footsteps

*Ji-Seok Kim^a,b,1^, Tae Woog Kang^c,d,1^, Hyunjoon Yoo^a^, Jawon Ha^a^, Yunuo Huang^d^, Hee Han^e^, Chi Won Ahn^e^, Woon-Hong Yeo^c,d,f,g*^, and Il-Kwon Oh^a*^*

a. Department of Mechanical Engineering, Korea Advanced Institute of Science and Technology (KAIST), 291 Daehak-ro, Yuseong-gu, Daejeon 34141, Republic of Korea
b. Department of Mechanical Engineering, Hanbat National University, 125 Dongseodae-ro, Yuseong-gu, Daejeon 34158, Republic of Korea
c. George W. Woodruff School of Mechanical Engineering, Georgia Institute of Technology, Atlanta, GA 30332, USA
d. Wearable Intelligent Systems and Healthcare Center (WISH Center) at the Institute for Matter and Systems, Georgia Institute of Technology, Atlanta, GA 30332, USA
e. National Nano Fab Center (NNFC), 291 Daehak-ro, Yuseong-gu, Daejeon 34141, Republic of Korea
f. Wallace H. Coulter Department of Biomedical Engineering, Georgia Institute of Technology and Emory University School of Medicine, Atlanta, GA 30332, USA
g. Parker H. Petit Institute for Bioengineering and Biosciences, Georgia Institute of Technology, Atlanta, GA 30332, USA

1 These authors contributed equally to this work.
* Corresponding authors (E-mail: whyeo@gatech.edu(W.H. Yeo), [ikoh@kaist.ac.kr(I.K.Oh)](mailto:ikoh@kaist.ac.kr(I.K.Oh)))

**Keywords:** wearable energy harvesters; smart shoes; PPG sensors; integrated biosensing; self-powered devices

**Figure S1. Gait characteristics during walking. a.** Schematic illustration of gait phases during walking. **b.** Heel pressure profiles of left and right feet. **c.** Plantar pressure distribution measured using the smart insole during walking.

**Figure S2. a.** Schematic illustration of plain rotors with different numbers of rotor blades. **b.** Rotational speeds of plain rotors.

**Figure S3. a.** Schematic illustration and **b.** photographs of a fabricated rotational electromagnetic generator (EMG).

**Figure S4. a.** Schematic diagram and **b.** photos of the experimental setup of the rotational EMG. **c.** Output voltage and **d.** frequency dependence on rotational speed to validate a linear proportional relationship. **e.** Output voltage profiles under different operating frequencies (2.5, 5, 9.8, and 14.71 Hz).

**Figure S5. a.** Schematic illustration of the testing environment for APEH performance evaluation **b.** Schematic diagram of gait phases and the corresponding plantar pressure profile. **c.** Output voltage profile of the APEH and representative local peak points. **d.** Temporal variation of voltage peak values during walking.

**Figure S6. a. Schematic illustration of experimental setup used to measure the heel-region pressure. Experimentally measured heel-pressure responses during walking b. without and c. with the air pump.**

**To clarify the pneumatic energy-buffering mechanism of the APEH, the transient heel-strike pressure was first measured with and without the air pump during walking as shown in Fig. S6. In both configurations, the flexible pressure sensor was placed on top of the insole, and each walking experiment was repeated five times at a walking speed of 3.5 km/h. Without the air pump, the heel region exhibited an abrupt impact response with a peak local pressure of approximately 450 kPa. After inserting the air pump, the peak pressure decreased to approximately 240 kPa, indicating that the compliant rubber bladder redistributed the heel-strike load and reduced the local impact pressure. In addition, the presence of the air pump prolonged the time required for the pressure response to reach its peak to approximately 0.3 s. This delayed pressure rise supports that the heel-strike impact was temporarily buffered by deformation of the rubber bladder and compression of the enclosed air before the stored pneumatic energy was released through the outlet as pressurized airflow. The stored pneumatic energy was then released as airflow through the nozzle to drive the rotor.**

**Figure S7. Schematic illustration of the energy flow and dynamic model of the air-pumping pneumatic energy harvester (APEH).**

Based on the observation, the APEH can be interpreted as a pneumatic compliant energy-buffering structure rather than a direct mechanical transmission system. **Fig. S7** schematically illustrates the energy-flow pathway and simplified dynamic model of the APEH. During heel strike, a partial portion of the mechanical work is transferred to the air pump and the mechanical input can be expressed as the follows:

$$W_{in}\approx\boldsymbol{\int}F_{pump}(x)dx=\boldsymbol{\int}F_{pump}(t)\dot{x}(t)dt,$$

where $F_{pump}$ is the effective compressive force acting on the air pump.

For a simplicity, the reaction force generated by the air pump can be considered to arise from two primary conditions:

$$F_{pump}\left( t \right)\approx F_{rubber}\left( x,\dot{x} \right)+A_{eff}\Delta p_{b}\left( t \right),$$

where $F_{rubber}\left( x,\dot{x} \right)$ represents the deformation resistance of the rubber bladder and $A_{eff}\Delta p_{b}\left( t \right)$ represents the force arising from the transient pressure difference between the bladder chamber and the ambient environment. The rubber-deformation term includes both the elastic restoring force and the rate-dependent viscoelastic resistance of the bladder. The pressure-induced term originates from compression of the enclosed air; however, the mechanical response of the air pump is governed not only by the material properties of the rubber bladder but also by the time-dependent pressure buildup and airflow release in the APEH system. The nozzle influences this process by regulating how rapidly the compressed air is discharged. Consequently, the nozzle geometry can alter the temporal evolution of the internal pressure, the deformation response of the bladder, and the time required for the pneumatic energy to be transferred to the rotor.

The pneumatic energy temporarily stored through air compression can be expressed conceptually as pressure–volume work:

$$E_{pneumatic}\approx-\int_{V_{i}}^{V_{f}} \Delta p_{b}dV_{b},$$

where *V_i_* and *V_f_* are the initial and compressed bladder volumes, respectively. The negative sign accounts for the decrease in bladder volume during compression. This stored pneumatic energy is subsequently released through the nozzle as pressurized airflow and converted into the rotational motion of the rotor.

This stored pneumatic energy is subsequently released through the nozzle as pressurized airflow and converted into the rotational motion of the rotor. The airflow exerts aerodynamic forces on the rotor blades, thereby generating a driving torque. The resulting rotor motion is determined by the balance between this airflow-induced torque and the opposing torques associated with the rotational system, including mechanical friction, aerodynamic losses, and the electromagnetic reaction torque generated by the coupled electromagnetic generator (EMG). As the rotor drives the EMG, electrical energy generation produces an electromagnetic damping ($\tau_{\mathrm{EM}}$) effect that resists the rotor motion. Therefore, the transient rotational response is governed by the combined effects of the supplied airflow, rotor inertia, aerodynamic interactions, and electrical loading of the EMG. Conceptually, the rotational dynamics can be expressed as

$$J\dot{\omega}\left( t \right)\approx\tau_{air}\left( t \right)-\tau_{loss}\left( t \right)-\tau_{EM}\left( t \right),$$

where $J$ is the rotational inertia of the rotor, $\omega$ is the angular velocity, $\tau_{air}$ is the airflow-induced driving torque, $\tau_{loss}$ represents the combined mechanical and aerodynamic losses, and $\tau_{EM}$ is the electromagnetic reaction torque associated with electrical energy generation.

**Figure S8. Comparison of voltage peak values to investigate the effect of nozzle parameters.** Schematic illustration of the experimental setup and voltage peak measured with the same area ratio and different nozzle lengths **a.** 5 mm, **b.** 15 mm, and **c.** 45 mm.

**Figure S9. Comparison of voltage peak values to investigate the effect of tube parameters**

Schematic illustration of the experimental setup and voltage peak measured with different tube curvatures **a.** 5.5 mm, **b.** 7.5 mm, and **c.** 10.0 mm.

**Figure S10. a.** Short-circuit current of APEH. **b.** Maximum and the RMS values of current under different load resistances. **c.** Maximum instantaneous and time-averaged power of APEH transferred to load resistances.

**Figure S11. Walking-speed-dependent APEH output characteristics. a.** APEH output voltage measured at different walking speeds. The step length was maintained constant to minimize the influence of heel-strike impact amplitude, while the walking interval between successive steps was reduced to increase the walking speed. **b.** RMS voltage with different walking speeds (1.75, 2.0, 2.78, and 3.5 km/h).

**Figure S12. Inter-subject APEH output comparison before and after walking-protocol training. a.** Initial APEH output voltage from multiple subjects with different body weights. The arrow inset indicates each footstep and the calculated RMS voltage and standard deviation are given in the inset of each waveform. **b.** APEH walking protocol for stable energy harvesting, including balanced weight loading at heel strike, full air-bladder compression during the stance phase, and passive reinflation from toe-off to the next heel strike. **c.** APEH outputs after 30 min of protocol training, showing substantially increased and comparable outputs across subjects.

In **Fig. S12**, we measured the APEH output voltage with multiple subjects with different body weights at the same speed (1.7 km/h) to investigate the effect of individual variability on the APEH performance. The initial output was not proportional to body weight, indicating that gait adaptation and effective air-bladder compression/reinflation are more important than body weight alone (**Fig. S12a**). The well-trained Subject A exhibited the highest RMS voltage of 0.5752 V, despite not having the highest body weight, whereas the heaviest subject showed the lowest RMS voltage of 0.2494 V. After 30 min of protocol training involving balanced weight loading at heel strike, full air-bladder compression by body weight during the stance phase, and passive reinflation from toe-off to the next heel strike as shown in **Fig. S12b**, the APEH outputs from subjects with different body weights increased substantially and became comparable (**Fig. S12c**). In conclusion, reliable APEH performance can be obtained across users after short familiarization with the in-shoe air-pump structure

**Figure S13. Flexible circuit design for a wearable ankle PPG device.**

**Figure S14. a.** Pulse rate of the wearable PPG device and the BioRadio, extracted from their PPG sensing. **b.** Correlation of pulse rate between the BioRadio and the wearable PPG device. Data in b were pooled from all 3 subjects and subsampled for visualization purposes; all statistical analyses were conducted on the complete dataset.

**Figure S15. Block diagram of PPG detection and SpO_2_ calculation algorithm.**

**Figure S16. a.** Pulse rate of the wearable PPG device, Sentec SDM, and the BioRadio (hallux), while standing and walking. **b.** Correlation of pulse rate between the Sentec SDM and other PPG devices. Data in b were pooled from all 3 subjects and subsampled for visualization purposes; all statistical analyses were conducted on the complete dataset.

**Figure S17. PPG raw signal-based venous reflux test with the wearable ankle PPG device.**

**Figure S18. a.** Schematic diagram of the validation test for the VM-based AC/DC converter. **b.** Original sinusoidal voltage signal from the function generator. **c.** Converted voltage signals using VM-based AC/DC converters with different capacitances. **d–e.** Schematic illustration of the working principle of the VM-based AC/DC converter.

**Figure S19. Direct battery charging using the VM-based AC/DC converter without applying the charger-IC current limit. a.** Schematic illustration of the power flow from footsteps to the battery. **b and c.** Charging current delivered to the battery under repeated footsteps using storage capacitances of 100 μF and 2,200 μF, respectively. Arrows and shaded regions indicate individual footsteps and pneumatic energy-buffering intervals, respectively. **d.** Footstep-averaged charging current delivered to the same battery for different storage capacitances in the VM-based AC/DC converter: 100, 470, 1,000, and 2,200 μF.

The direct battery-charging behavior of the VM-based AC/DC converter was evaluated without applying the charger-IC current-limiting threshold as shown in **Fig. S19**. Because the APEH generates pulsed electrical output under repeated footsteps, the storage capacitance strongly affects transient charge transfer to the battery. With a small capacitance of 100 μF, the converter voltage rapidly increases under pulsed excitation, resulting in large current fluctuations and occasional negative current components. As the capacitance increases, the charging current becomes more stable, and excessive transient peaks are suppressed. These results indicate that increasing the storage capacitance in the VM-based AC/DC converter can stabilize battery-charging behavior under intermittent gait excitation. However, excessively large capacitors increase the physical size of the power-management circuit; therefore, the capacitance should be selected by considering both charging stability and the compact form factor required for wearable integration.

**Figure S20.** a. Block diagram of the charger IC used for current control. (b) PCB layout of the battery charger.

**Figure S21. Battery charger IC current-limiting test using a controlled DC power supply. a.** Schematic diagram of the measurement setup, in which the power supply input voltage and supply-side current setting were varied under constant-voltage (CV) operation, and the battery-side current was measured using an ammeter. **b.** Battery charging current measured under different input conditions.

The current-limiting function of the battery charger IC was verified using a controlled DC power supply as shown in **Fig. S21**. The input voltage and supply-side current setting were varied under constant-voltage operation, and the battery-side current was measured using an ammeter. When the supply-side current setting was 1 mA, intermittent charge transfer was observed under marginal input-current conditions, indicating repeated enabling and interruption of charge transfer by the charger IC. When the supply-side current setting was increased to 2 mA, the battery-side current was regulated at approximately 1 mA over the tested voltage range. These results confirm that the charger IC effectively limits the current delivered to the battery according to the programmed current-limiting threshold. Therefore, the 1 mA value used in the manuscript should be interpreted as the maximum allowable charging-current threshold of the charger IC, rather than the long-term average charging current generated by the APEH system.

**Figure S22. Changes in pulse rate of a subject wearing the energy-harvester-integrated wearable ankle PPG platform during daily routine.**

**Figure S23. Demonstration setup for PPG monitoring while sleeping.**

**Figure S24. Pulse rate of the wearable PPG device, Sentec SDM, and the BioRadio (index finger), while sleeping.**

**Table S1.** Demographic information and device placement of subjects enrolled in the feasibility study

| **Subject** | **Photo** | **Age** | **Sex** | **Fitzpatrick Skin Type** | **BMI**  **(kg/m²)** |
| --- | --- | --- | --- | --- | --- |
| 1 |  | 35 | Male | IV | 22 |
| 2 |  | 33 | Female | III | 25 |
| 3 |  | 30 | Male | IV | 20 |

**Table S2.** Comparison of shoe-mounted energy harvesters based on practical output metrics and dimensions of the integrated system

| **Ref.** | **Energy harvesting technology** | **Device size[mm^3^] /mass [g]** | **Walking speed**  **[km/h]** | **Subject weight  [kg]** | **Load condition** | **Time-averaged output power [mW]** | **Energy delivered to storage per step [mJ]** | **Averaged power to storage per unit device mass [mW/kg]** |
| --- | --- | --- | --- | --- | --- | --- | --- | --- |
| [1] | Rotational triboelectric (FEP/Al) | 70 x 35 x 131 / 200 | 3.5 | N.A | Exo-shoe lever | N.A | 0.11* | 0.55 |
| [2] | Piezo-electric (PZT) | 40 x 60 x 0.2 / N.A | N.A | 60  70  80 | Insole | N.A | 0.006*  0.011*  0.013* | N.A |
| [3] | Piezo-electric (PZT) | 45 x 30 x 24 / 22.3 (frame only) | 2  4 | 65 | Magnetic excitation + cantilever  under the Insole | 0.18 | 0.03  0.18 | N.A |
| [4] | Electro- magnetic + triboelectric | 40 x 55 x 0.2 (Tribo), 764.5 (EMG) / N.A | N.A (1 Hz) | 70 | Aeroelastic structure | N.A | 0.3* | N.A |
| [5] | Electro- magnetic | 70 x 43 x 25 / 55 | N.A  (1 Hz) | N.A | Ratchet –based frequency-upconversion | 0.12 | N.A | N.A |
| **This work** | **Electro- magnetic** | **Diameter: 55 under the insole (inner), Diameter: 50, thickness: 30 (rotor/EMG)/ 125** | **3.5** | **70** | **Pneumatic energy buffering (insole)** | **1.93** | **0.93** | **7.44** |

*Estimated from reported capacitor-charging curves or step-energy data.

**References**

[1] Y. Yun, S. Jang, S. Cho, S. H. Lee, H. J. Hwang, and D. Choi, “Exo-Shoe Triboelectric Nanogenerator: Toward High-Performance Wearable Biomechanical Energy Harvester,” Nano Energy 80 (2021): 105525, https://doi.org/10.1016/j.nanoen.2020.105525.

[2] S. Y. Jeong, L. L. Xu, C. H. Ryu, et al., “Wearable Shoe-Mounted Piezoelectric Energy Harvester for a Self-Powered Wireless Communication System,” Energies 15, no. 1 (2022): 237, https://doi.org/10.3390/en15010237.

[3] K. Fan, Z. Liu, H. Liu, L. Wang, Y. Zhu, and B. Yu, “Scavenging Energy from Human Walking through a Shoe-Mounted Piezoelectric Harvester,” Applied Physics Letters 110, no. 14 (2017): 143902, https://doi.org/10.1063/1.4979832.

[4] Y. Gao, R. Zong, J. Feng, Y. Li, Z. Chen, and L. Qi, “Flexible Self-Powered and Self-Sensing Shoes Based on Aeroelastic Structure for Application in Human Motion Monitoring,” Journal of Power Sources 638 (2025): 236626, https://doi.org/10.1016/j.jpowsour.2025.236626.

[5] Z. Yin, S. Gao, L. Jin, S. Guo, Q. Wu, and Z. Li, “A Shoe-Mounted Frequency up-Converted Piezoelectric Energy Harvester,” Sensors and Actuators A: Physical 318 (2021): 112530, https://doi.org/10.1016/j.sna.2020.112530.
